# Does the Public Telephone Transmit Disease?1Read before the Medical Union, December 17, 1902.

**Published:** 1903-01

**Authors:** William G. Bissell

**Affiliations:** Buffalo, N. Y.; Bacteriologist, Department of Health


### Does the Public Telephone Transmit Disease?[1]

By WILLIAM G. BISSELL, M. D., Buffalo, N. Y.

Bacteriologist, Department of Health.

VARIOUS manufacturers have, from time to time, turned out devices to be attached to the transmitters of public telephones, claiming that their use minimises the possible transmission of disease from this source. The question often has been asked, "Does the public telephone transmit disease"?

In order to discuss this question, two important points must be considered:

1. Read before the Medical Union, December 17, 1902.

First, is the human body, and particularly the mouth, of well persons, a possible carrier of infection?

Second, if so, what part does the telephone play in transferring such infection?

The popular conception that a disease germ enters the body, produces its disease, and passes out again without receiving a great amount of resistance, is not confirmed by scientific investigation and experiment. In every infection there are two opposing forces, and the variation in the resistance of the individual and the strength of the germ decides the resulting factor.

If the germ has the greater power, the disease is the result, but if the vital forces are in excess, the organism, although it may have gained entrance, does not cause disease. The same is true of the final exit and the destruction of the germ after the recovery from disease; and this leads to the inference, proven in a variety of conditions, that persons, to all appearances physically well, may become carriers of germs. Experimental evidence has shown that many germs carried under these conditions are capable of infecting animals, and there is no basis for belief that germs carried in this way are none other than harmful. Their harmlessness in the instance mentioned simply expresses the degree of immunity, either inherited or acquired, to the particular disease represented by the germ.

Laboratory tests clearly demonstrate that microbes identified as belonging to a particular species of pathogenic bacteria are, as a rule, virulent. Such organisms, when carried in different locations, are capable of being shed, hence constitute a source of danger in the transmission of disease. For example, in chronic pulmonary tuberculosis, the disease being one of the respiratory tract, and invariably accompanied by coughing, the organism is expelled in immense numbers. Individuals who have recovered from typhoid fever and cholera, although not ill, continue to give off the germs of these respective diseases.

It is probable that the microbe of influenza vegetates on the mucous membranes for a period of time after recovery from that disease. It has been known, for more than twenty years, that the germ of croupous pneumonia flourishes in the mouth of every healthy person. If we consider these facts, and remember that the diphtheria bacillus may, and very frequently does, persist in the throat and nose of persons who have come in contact with diphtheria, and yet are clinically well, it makes one believe that, by the use of the telephone, on account of the close proximity of the mouth, it is possible for infection of various kinds to be deposited upon the transmitter, and that persons, during inhalation while using the instrument, might become infected.

In order to positively ascertain if such conclusion was correct, during the early part of the present year, experiments were inaugurated along the following line: statistics show that the diphtheria bacillus is contained in the throat of about one person in each one hundred. If this be true, and there is no reason to doubt the accuracy of the statement, it is fair to suppose that telephones situated in such localities as the Hotel Iroquois, the Genesee Pharmacy, the Tifft House and the Genesee Hotel, would, during a period of constant use, become infected with this organism.

The telephones in these localities are used by many hundreds of persons during each day, and in view of this fact, cultural examinations were made as follows:

Twenty-five cultures were taken, using sterilised swabs, the same being rubbed around the entire inner surface of the transmitters. At the Hotel Iroquois, such investigation covered a period of four different days; at the Tifft House, three different days; at the Genesee Pharmacy, two different days; and at the Genesee Hotel, one day. It is fair to assume that, if the diphtheria bacillus was contained in the transmitters, a procedure of this character would demonstrate its presence. After most persistent search, it was impossible to demonstrate the presence of this organism.

It must be remembered, however, that the telephones in the locations mentioned, are kept in a cleanly manner, the transmitters being frequently wiped by the use of a dampened cloth. In view of these circumstances, it is fair to assume that by the use of telephones which are kept ordinarily free from dust, there is little danger of the transmission of disease.

---

### ACUTE BRONCHITIS.

THIS prescription, credited in *Le Progres Medicale* to G. Lyon, appears to be a very good mixture for acute bronchitis :

| | | | |
|---|---|---|---|
| ℞ | Tinct squills | 0.7 | m x. |
| | Syrup codeia | 20.0 | ʒ v. |
| | Ext. hyoscyamus | 0.06 | gr. i. |
| | Orange-flower water | 10.0 | ʒ ii. ss. |
| | Julep | 120.0 | ℥ iv. |

Mix. Dose—One dessertspoonful.

